# Circadian rhythm and surface activity in soil-dwelling caecilians (Amphibia: Gymnophiona)

**DOI:** 10.1038/s41598-024-60533-5

**Published:** 2024-04-30

**Authors:** Avanthika Prakash, David J. Gower, Ranjith Vengot, Ramachandran Kotharambath

**Affiliations:** 1 Department of Zoology, Central University of Kerala, Tejaswini Hills, Kasaragod, Kerala India; 2 Natural History Museum, London, SW7 5BD UK

**Keywords:** Animal behaviour, Herpetology

## Abstract

The degree to which burrowing, soil-dwelling caecilian amphibians spend time on the surface is little studied, and circadian rhythm has not been investigated in multiple species of this order or by manipulating light–dark cycles. We studied surface-activity rhythm of the Indian caecilians *Ichthyophis* cf. *longicephalus* and *Uraeotyphlus* cf. *oxyurus* (Ichthyophiidae) and *Gegeneophis tejaswini* (Grandisoniidae), under LD, DD and DL cycles. We examined daily surface activity and the role of light–dark cycles as a zeitgeber. All three species were strictly nocturnal and *G. tejaswini* displayed the least surface activity. Four out of thirteen individuals, two *I.* cf. *longicephalus*, one *G. tejaswini* and one *U.* cf. *oxyurus*, displayed a more or less distinct surface-activity rhythm in all three cycles, and for the nine other animals the activity patterns were not evident. An approximately 24 h free-run period was observed in the three species. When the light–dark cycle was inverted, surface activity in the three species shifted to the dark phase. The findings of this study suggest that caecilians have a weak circadian surface-activity rhythm, and that the absence of light can act as a prominent zeitgeber in these burrowing, limbless amphibians.

## Introduction

Circadian rhythm in animals exposed to little or no light is a topic of broad research interest. Circadian rhythm is a ubiquitous phenomenon controlled by endogenous oscillators entrained to an environmental cue, the zeitgeber^1^. One prominent zeitgeber is the natural photoperiod. In most animals, this reliable, cyclic, light–dark cycle acts as an environmental reference point and entrains the daily physiological and behavioural rhythm. Experiments on terrestrial, burrowing adults of a single species of caecilian (elongate, limbless amphibians of the Order Gymnophiona), identified that they respond to the transition of light and dark phases by using light as the zeitgeber to synchronize activities on a day-night rhythm [2, and references reported therein]. The animals maintained their normal rhythm when the eyes were surgically removed, showing that they also use extraocular systems, including the pineal system, to regulate their surface activity^2^.

Most circadian rhythm studies in vertebrates living in dark environments have been carried out on burrowing rodents such as mole rats^3–5^ and cave fishes^6–8^. Despite being strictly subterranean and characterized by regressed visual systems, all mole-rat species studied so far have functional circadian systems with varying degree of rhythmicity^9^. Many of these species exhibit endogenous locomotor activity rhythm entrained to the 24 h light–dark cycles^10–12^, and dawn and dusk are crucial environmental cues in generating the circadian pattern of locomotor activity in rodents^13^. Also, mole rats display large inter- and intra-specific variation in locomotor activity rhythm^14–16^. A regression of circadian locomotor activity has been reported in some cave fishes^17,18^ and cave salamanders^19^ that have reduced visual systems. These studies suggest the presence of circadian rhythm in many animals occupying even very dimly lit environments, though sometimes with environmental cues other than photoperiod acting as the zeitgeber. However, making generalisations and explaining exceptions, such as whether regression of circadian rhythm is explained by, for example, extent and/or age of light-avoiding behaviour, requires additional studies of other lineages that inhabit low- or zero- light environments. Barring the above-mentioned observations of a single species without manipulating light–dark cycles^2^, there have not been any studies on the interplay between natural light and activity in caecilians, even though they are a potentially interesting group of lower vertebrates for studying circadian rhythms because of their disparity, long evolutionary history and ecomorphological diversity^20^.

**Figure 1:**
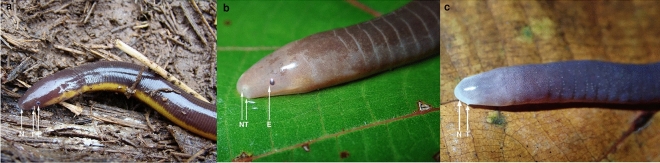
Photos of the three study species: (**a**) *Ichthyophis longicephalus,* (**b**) *Uraeotyphlus* cf. *oxyurus* and (**c**) *Gegeneophis tejaswini.* Arrows indicate position of eye (E: when visible externally), nostril (N) and tentacle (T).

The general caecilian phenotype reflects their fossoriality, with adaptations such as lack of limbs and girdles, compact heavily ossified skulls^20^, highly reduced eyes that are covered by skin or bone, possibly rod-only retinas^21^, and the presence of a pair of sensory tentacles^22^. Sensory tentacles are unique to caecilians, and the other features are not found in combination in the other dark-environment vertebrates in which circadian rhythm has been studied. Adults of most caecilian species (including the basalmost lineages) are terrestrial burrowers in tropical soils, but their ecology and behaviour are little-studied and generally poorly known^23^. Although rarely studied quantitatively or experimentally, encounters of caecilians in the field and variation in aspects of their morphology (e.g., degree of skin pigmentation and reduction of the visual system; position of tentacles: e.g., Fig. 1) suggest notable interspecific variation in the degree of fossoriality and surface activity^20^. Caecilians use ocular and extraocular systems for photoreception^2,24^, and use air- and surface-borne chemicals brought into the nasal cavities via the nostrils and tentacles to additionally navigate and feed^25^ and perhaps locate mates in their mostly dim-lit or dark soil habitats. Better understanding of circadian rhythms (and surface-activity patterns) in caecilians will not only contribute to a more complete understanding of vertebrate circadian rhythms, but will also help to better design and interpret data generated from quantitative ecological surveys of caecilians—sorely needed to improve knowledge of the conservation requirements and natural history of these generally poorly known vertebrates^23^.

Here we present the results of an experiment conducted under various light–dark cycles in controlled laboratory conditions on three caecilian species from the Western Ghats of peninsular India. This is the first investigation of circadian rhythm and surface activity in Indian caecilians, the first study of caecilian circadian rhythms that includes manipulation of light–dark cycles, and the first comparative study of these aspects of the biology of Gymnophiona.

## Methods

### Animals

Thirteen adult specimens of three caecilian species (Fig. 1) were collected from north Kerala, part of the Western Ghats region of peninsular India. Sampling comprised five *Uraeotyphlus* cf*. oxyurus* (Kannur and Kasaragod: total length 215–272 mm) and three *Ichthyophis* cf. *longicephalus* (Kasaragod: 217–240 mm) (Ichthyophiidae) and five *Gegeneophis tejaswini* (Kasaragod: 130–172 mm) (Grandisoniidae). These taxa were selected to include the three caecilian genera of the southern Western Ghats, and the three particular species were chosen based on local availability. Animals were maintained in a husbandry facility at 23–25 °C, kept in ventilated plastic boxes containing a moist, pulverized coir substrate, and fed with live earthworms. Substrate and diet were chosen based on previous preliminary data on its suitability, and temperature was based on similar data from the collection sites of the experimental animals. The husbandry facility receives daylight through a window, providing the animals with a natural 12L/12D photoperiod. The sex of the animals could not be identified, because the study species are not obviously sexually dimorphic externally. All procedures were approved by the Institutional Animal Ethics Committee (IAEC), Central University of Kerala, India. All experiments were carried out in accordance with the ethical guidelines and regulations of the Committee for the Purpose of Control and Supervision of Experiments on Animals (CPCSEA, India) and in accordance with ARRIVE guidelines.

### Experimental design

Each animal was housed separately in a plastic box (29.8 × 22.7 × 13.2 cm), one-third of which was filled with moist coir. The box was placed in a dark room within the husbandry facility, maintaining an ambient temperature of 23–25 °C. Room temperature and relative humidity were monitored continuously using a data logger (Tinytag, UK). Four 3 W and one 5 W LED bulbs were used to light the room during the LD and DL cycles.

The experimental light regimes comprised three light–dark cycles. The animals were initially exposed to 12L/12D condition (LD) for four days, followed by constant dark (DD) condition for ten days, and then an inverse photoperiod of 12D/12L (DL cycle) for five days. Light intensity (measured using an LX-101A Light Meter, HTC) varied from 150–230 lx during the light phase to 1.3 lx during the dark phase of the cycles. For each cycle, surface activity of the animals was monitored and recorded using three night-vision cameras (Yale HD1080) positioned above the animal boxes. Animals were considered “surface active” if any part of the animal was observed on the surface of the substrate, whether moving or lying inactive on the surface.

### Data analysis

Video recordings were analysed manually using the VideoLAN Client (VLC) (version 3.0.16) media player under the default motion detection function. The time of arrival and withdrawal of the animal on the surface was noted, and the data were arranged in 30 min intervals for analysis. Double-plotted actograms representing the daily surface activity of the animals were plotted in Actogram J software version 1.53^26^. Normalized average surface activity of individuals was represented as the actogram of each species. The period of the endogenous rhythm was evaluated by Lomb-Scargle periodograms generated by the automatic period detection tool in Actogram J, using data from 10 consecutive days in DD cycle with a significance level of *p* < 0.05. Rose plots were used to visualize activity time period and were generated using RhythmicAlly^27^. All statistical comparisons were analyzed using the Kruskal–Wallis test followed by post-hoc analysis using the Dunn test, and the *p* value was adjusted by the Holm method. Statistical analyses were performed using R (version 4.2.2) and *p* values < 0.05 were considered statistically significant. Average values are expressed as mean ± standard error (SE). Duration of activity episodes is shown as median values.

## Results

### General surface-activity patterns

**Figure 2:**
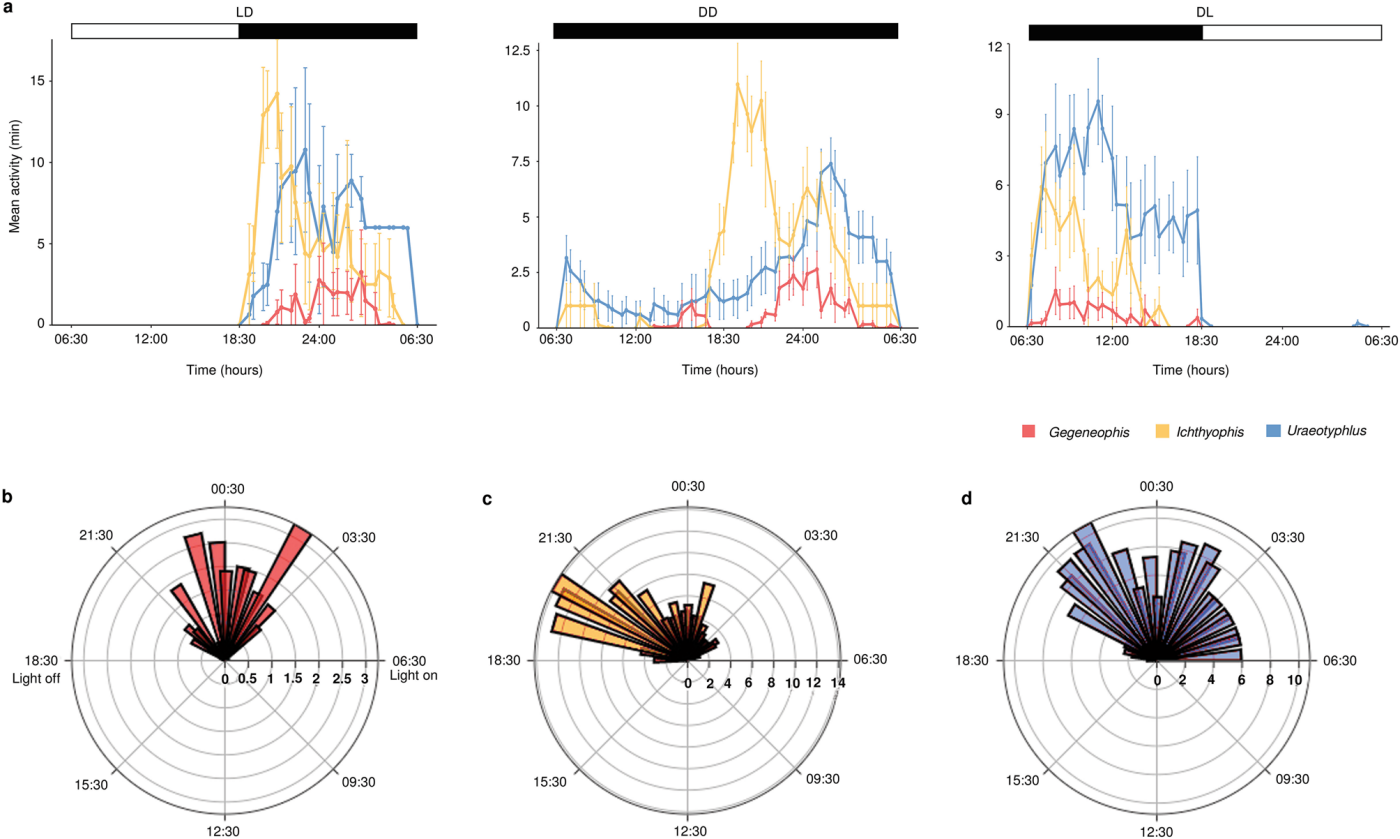
General surface-activity pattern of three caecilian species. (**a**) Mean activity of individuals of each species displayed in 30 min bins under LD, DD and DL cycles are plotted against time in 24 h. Values are shown as mean ± SE. (**b**–**d**) Circular histogram (rose plot) of the surface activity of (**b**) *Gegeneophis tejaswini*, (**c**) *Ichthyophis* cf. *longicephalus* and (**d**) *Uraeotyphlus* cf. *oxyurus* displayed in LD cycle. Mean surface activity is represented (in minutes) on the radial axis and time in 24 h is denoted on the outer circle.

All three study species displayed a strictly nocturnal surface-activity pattern (Fig. 2a). Overall, there were differences among species in mean daily surface activity, duration of each episode of surface activity, and activity period. Mean daily surface activity was significantly greater in *I.* cf*. longicephalus* and *U.* cf. *oxyurus* than in *G. tejaswini* (111.77 ± 16.11 min and 130.63 ± 19.51 min vs. 20.68 ± 3.73 min, *p* < 0.01), but not significantly different between *I.* cf. *longicephalus* and *U.* cf. *oxyurus.* Most (65.2%) of the surface activity of *G. tejaswini* was in episodes of less than five minutes, whereas *U.* cf. *oxyurus* undertook fewer surface-active episodes of less than five minutes (37.8%), and among the episodes of more than five minutes, there were more (17%) of durations more than one hour. Most (78.2%) of the surface-active episodes of *I.* cf. *longicephalus* were shorter than 30 min, with few (7.3%) episodes of more than one hour.

The three species displayed a striking difference in the onset and offset of surface activity. Under the standard 12L/12D photoperiod, surface activity of *G. tejaswini* occurred between 20:00 and 04:57, with neither the activity onset nor offset coinciding with the start and end of the dark phase (Fig. 2b). *Ichthyophis* cf. *longicephalus* was surface active at the start of the night, with the activity onset coinciding closely with the start of the dark cycle, and the activity offset occurred before the end of the dark phase (Fig. 2c). *Uraeotyphlus* cf. *oxyurus* were surface-active throughout the dark phase, with variation in the onset and offset (Fig. 2d).

### Activity in LD, DD and DL cycles

**Figure 3:**
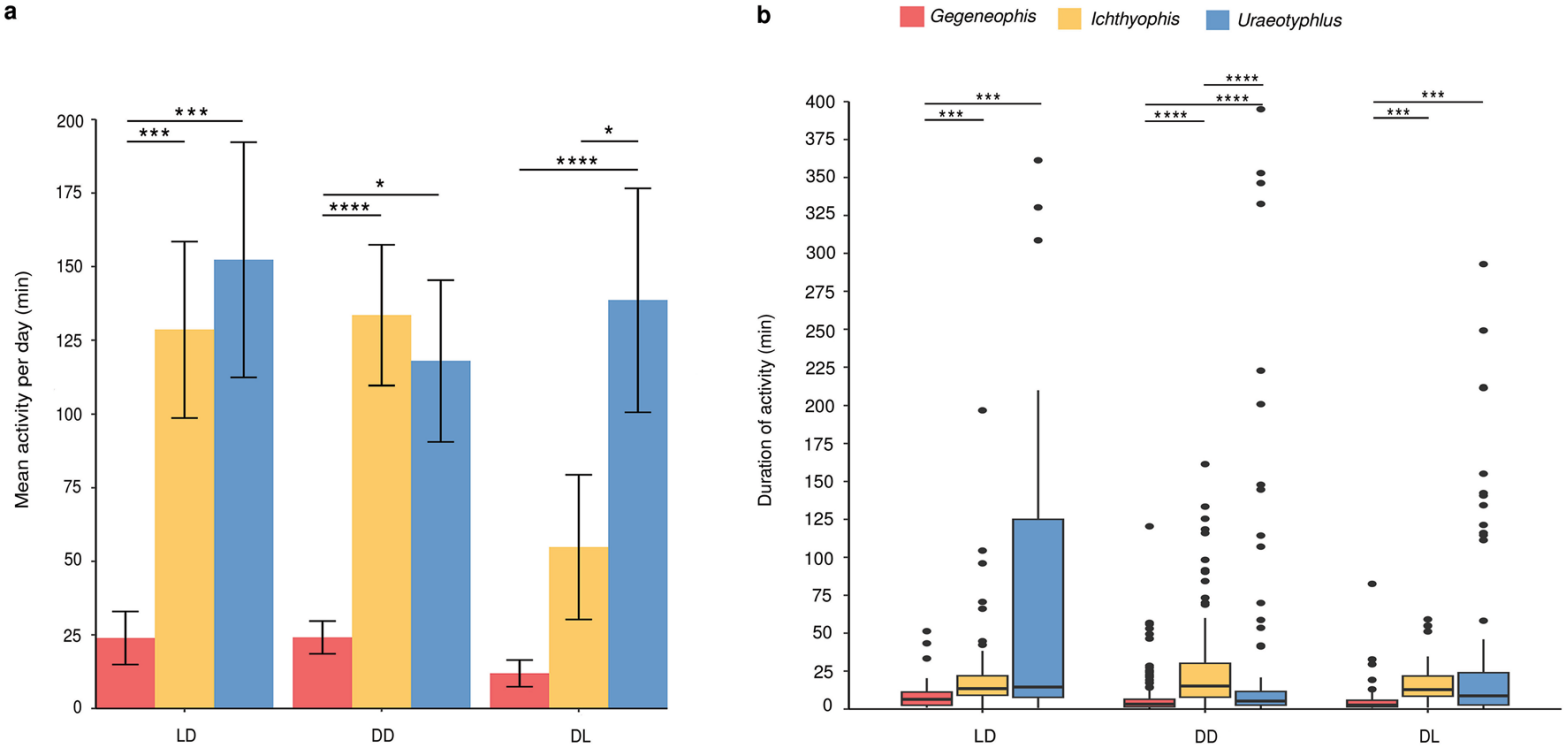
Comparison of mean surface activity and individual surface-activity episodes of three caecilian species in LD, DD and DL cycles. (**a**) Mean daily activity of individuals of *Gegeneophis tejaswini* (n = 5), *Ichthyophis* cf. *longicephalus* (n = 3), and *Uraeotyphlus* cf. *oxyurus* (n = 5) in three cycles. Values are shown as mean ± SE. (**b**) The box plot represents the duration of activity episodes in the three species. **** *p* < 0.001, ***p < 0.01 and *p < 0.05. Statistical comparison of mean activity of species and duration of activity of species were undertaken using Kruskal–Wallis with a post-hoc Dunn test.

The mean daily total of surface activity and duration of each surface-active episode differed among the three species in the LD, DD and DL cycles. In LD and DD cycles, mean surface activity of *U.* cf. *oxyurus* and *I.* cf. *longicephalus* was significantly greater than *G. tejaswini* (LD: 152.31 ± 40.99 min and 128.59 ± 31.24 min vs. 23.91 min ± 9.25 min, *p* < 0.01; DD: 117.97 ± 27.67 min and 133.52 ± 24.27 min vs. 24.13 ± 5.62 min, *p* < 0.001 and *p* < 0.05 respectively), but not significantly different between *I.* cf. *longicephalus* and *U.* cf. *oxyurus*. In DL cycle, *U.* cf. *oxyurus* displayed more surface activity than *G. tejaswini* (138.61 ± 38.77 min vs. 11.19 min ± 4.62 min, *p* < 0.001) and *I.* cf. *longicephalus* (138.61 ± 38.77 min vs. 54.82 min ± 25.43 min, *p* < 0.05) (Fig. 3a). Additionally, surface activity of *G. tejaswini* consisted of shorter episodes than in the other two species (LD: 6.4 min vs. 13.40 and 14.50 min, *p* < 0.01; DD: 3.20 min vs. 15.20 and 5.40 min, *p* < 0.001; DL: 2.6 min vs. 12.80 and 8.95 min, *p* < 0.01), with surface-active episodes not differing significantly between *U.* cf. *oxyurus* and *I.* cf. *longicephalus* in LD and DL cycles. However, in DD cycle, *I.* cf. *longicephalus* undertook longer episodes of activity than *U.* cf. *oxyurus* (15.20 min vs. 5.40 min, *p* < 0.001) and *G. tejaswini* (15.20 min vs. 3.20 min, *p* < 0.001) (Fig. 3b).

In summary, across all three light cycles, *G. tejaswini* displayed the least amount of surface activity and with shorter episodes of activity, with the other two species not differing significantly in mean surface activity under LD and DD cycles. Mean surface activity did not differ significantly across the three light cycles in *G. tejaswini* (*p* = 0.58) and *U.* cf. *oxyurus* (*p* = 0.57). In *I.* cf. *longicephalus,* surface activity did not differ between LD and DD cycles (*p* = 0.88) but was significantly reduced under the DL cycle (54.82 ± 25.43 min in DL vs. 128.59 ± 31.24 min in LD, *p* < 0.05; 54.82 min ± 25.43 min in DL vs. 133.52 ± 24.27 min in DD, *p* < 0.05) (Fig. 3a).

### Effect of light–dark cycle on surface activity

**Figure 4:**
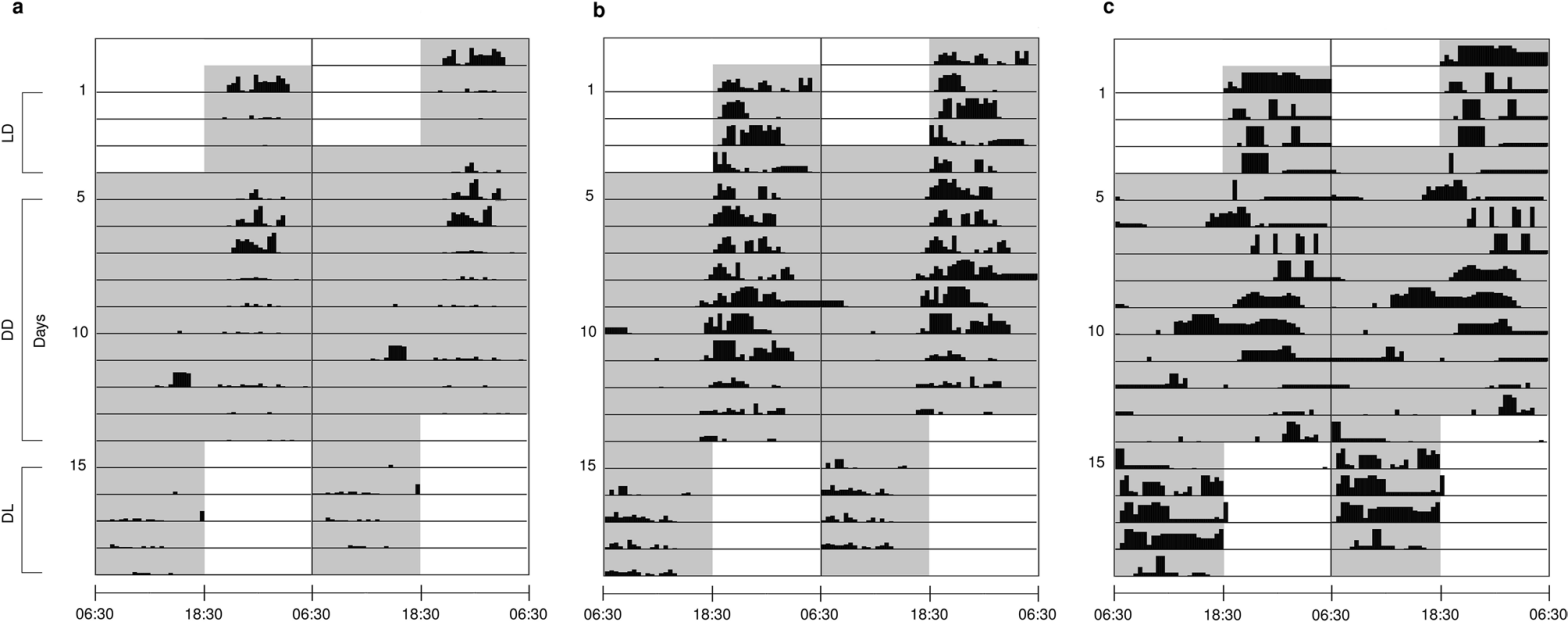
Surface-activity pattern of three species in LD, DD and DL cycles. Double-plotted actograms represent the normalized mean activity of individuals of (**a**) *Gegeneophis tejaswini* (n = 5), (**b**) *Ichthyophis* cf. *longicephalus* (n = 3) and (**c**) *Uraeotyphlus* cf. *oxyurus* (n = 5)*.* Activity was normalized in Actogram J using a reference actogram. Actograms with the greatest activity were used as the reference actogram for each species. The white and gray background indicates the light and dark phases, respectively, in three cycles.

**Figure 5:**
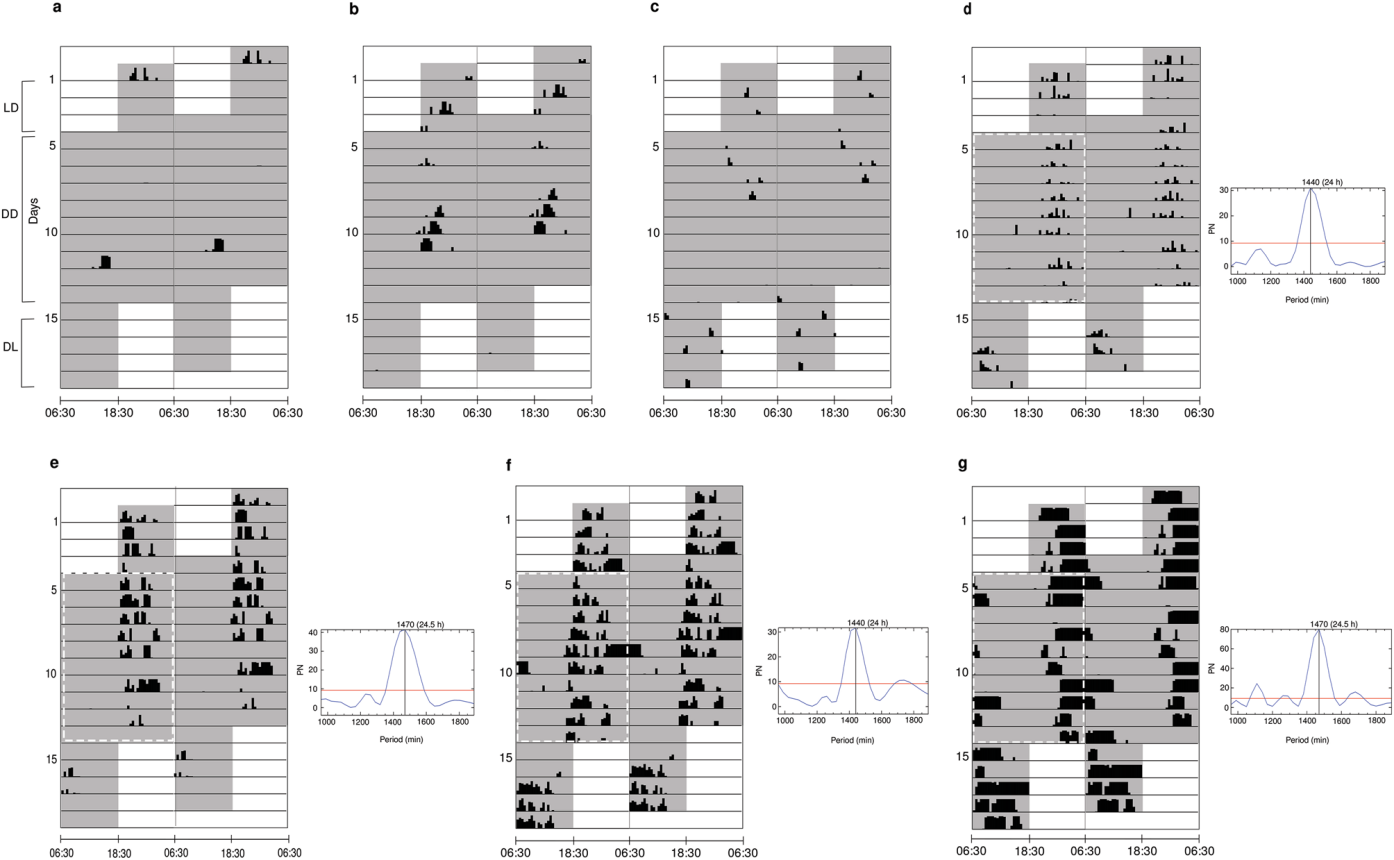
Double-plotted actograms of seven individuals showing surface-activity patterns of (**a**,**d**) *Gegeneophis tejaswini*, (**b**,**e**,**f**) *Ichthyophis* cf. *longicephalus* and (**c**,**g**) *Uraeotyphlus* cf. *oxyurus.* (**a–c**) Individuals with no evident surface-activity rhythm, and (**d**–**g**) individuals with evident surface-activity rhythm and corresponding Lomb-Scargle periodograms. The white and gray background indicates the light and dark phases, respectively, in three cycles. Dashed white lines illustrate the number of days used to determine the free-running period. The value of the peak periodogram amplitude in minutes and hours is denoted on the top of the corresponding peak. The red line in the periodograms indicates the significance level (*p* < 0.05).

Surface activity of the three species was restricted to the dark phase of the cycles, except for one incidence (as explained later, and shown in Fig. 4), and the activity pattern shifted in sync with light–dark cycles (Fig. 4). Four out of thirteen individuals, two *I.* cf. *longicephalus*, one *G. tejaswini* and one *U.* cf. *oxyurus*, displayed a more or less distinct surface-activity rhythm in all three cycles (Fig. 5). For the nine other animals the activity patterns were not evident (Supplementary Fig. S1). In LD cycle, these individuals displayed a nocturnal surface-activity rhythm and, as summarised above, the active period was 18:46–06:30 h for *U.* cf. *oxyurus* but 18:30–05:15 h and 20:09–04:57 h for *I.* cf. *longicephalus* and *G. tejaswini,* respectively. Among the three species, *U.* cf. *oxyurus* exhibited more inter- and intra-individual variation in the time of onset and offset of activity (Supplementary Fig. S1).

In DD cycle, the four active individuals exhibited a moderately evident endogenous rhythm with a period close to 24 h. The free-run period (the time required for the circadian rhythm to occur in constant conditions^28^) was 24 h in *G. tejaswini*, 24.5 h in *U.* cf. *oxyurus*, and 24 and 24.5 h in the two *I.* cf. *longicephalus* (Fig. 5d–g). Other individuals displayed a less-evident surface-activity rhythm and their onset and offset of activity varied irregularly (Fig. 5a–c; Supplementary Fig. S1). *Gegeneophis tejaswini* and *I.* cf. *longicephalus* followed a similar activity pattern to that which they displayed in LD cycle. A drift in surface activity to the subjective day occurred at the earliest from Day 5 and 6 of the DD cycle in *I.* cf. *longicephalus* and *G. tejaswini,* respectively*.* In contrast, surface activity of *U.* cf. *oxyurus* drifted into the subjective day on the first day of the DD cycle (Fig. 4), and there was substantial inter- and intra-individual variation in the activity duration and the time of onset and offset of activity in the latter species.

During DL cycle, the surface activity of all animals was restricted to the dark phase of the cycle, except for *U.* cf. *oxyurus* which was surface active for a short duration (12.1 min) in the light phase of the DL cycle. The inversion of the light–dark cycle caused a shift in the surface-activity pattern of the three species to the dark phase of the cycle*.* All five individuals of *U.* cf. *oxyurus *were surface active in the DL cycle, but only one displayed an evident pattern where onset of surface activity coincided with the start of the dark phase. The remaining individuals showed large inter- and intra-individual variation in the onset and offset of activity. In *G. tejaswini*, two of the five individuals showed a surface-activity pattern similar to the LD cycle activity. Similarly, two of the three *I.* cf. *longicephalus *showed a surface-activity pattern identical to that observed in the LD cycle. Also, there was a considerable reduction in the surface activity of two *I.* cf. *longicephalus* in DL compared to LD and DD (Supplementary Fig. S1).

## Discussion

The results of our experiments indicate that the three caecilian species investigated have a weak circadian rhythm in surface activity, with the absence of light acting as a zeitgeber. The three study species are all nocturnal in their surface activity, with *Gegeneophis tejaswini* being much less surface-active than the two ichthyophiid species studied. The major interspecific differences match expectations based on general observations of these animals in the lab and field. In our experience, species of *Gegeneophis* are typically found only by actively digging in soil, whereas adults of Indian ichthyophiids can occasionally also be encountered on the surface, particularly at night and/or during or soon after heavy rain. The major interspecific differences in degree of surface activity also largely match general understanding of caecilian ecomorphology^20^, wherein features such as more substantially reduced pigmentation and eyes, a closed orbit, more cylindrical body, and less protrusible tentacles (such as occur in *G. tejaswini*: Fig. 1)^29,30^ are considered generally to correlate with a greater degree of fossoriality. A notable finding of our experiments was intraspecific variation in the extent of surface activity, with some individuals active in all cycles and others generally preferring to stay within the substrate. The most surface-active individuals were active across all of the experimental cycles. The identification of individualistic expression of surface-active behaviour versus a rigid species-specific behaviour in these animals is a matter of further research.

With respect to the circadian rhythms observed in this study, caecilians generally resemble species of fossorial mole rats that have moderately reduced visual systems, rather than those with more reduced visual systems that lead a more dedicated subterranean existence for which other environmental cues, such as ambient temperature (e.g.,^9^), are the primary zeitgeber for more regressed circadian rhythms. Some non-mammalian vertebrates with strongly reduced visual systems lack notable circadian rhythms, but these live in largely arhythmic cave environments rather than burrow in soil^9^.

Circadian rhythms are adaptive in rhythmic environments^31^, including soils, so it is not unexpected that caecilians have intact rhythms despite their fossoriality, or that natural photoperiod is retained as a zeitgeber given that these amphibians are somewhat periodically active at the surface.

Nocturnality in the studied caecilians might be explained by various factors, including one or more of avoidance of bright light and/or predators, dispersing during cooler and more humid conditions, or finding prey and/or mates. More research is required to test such hypotheses because very little is known about aspects of caecilian biology such as behaviour, sociality or trophic relations.

The main limitation of our study (other than sample size) is that circadian rhythm was investigated only in terms of surface activity. It remains unknown whether these or other caecilian species display circadian rhythms also in their within-soil activity. Other avenues worthy of future research that could build on the present study include investigations that also take into account the effect of substrate type and ambient conditions on activity patterns. It is known from both field^32,33^ and laboratory^34,35^ studies that caecilians vary in their seasonal activity and have substrate preferences. Such research would also help to design further chronobiological experiments on caecilians. The present study adds to the small but growing body of work demonstrating that observations of caecilians in captivity can contribute insightful quantitative data to the understanding of the ecology and behaviour of these challenging study taxa (e.g.,^25,34–38^).

### Supplementary Information

Supplementary Figure S1.

## Data Availability

The datasets generated during and/or analysed during the current study are available from the corresponding author on reasonable request.
