# Supplementary material for: Circadian rhythm and surface activity in soil-dwelling caecilians (Amphibia: Gymnophiona)
Source: Sci Rep. 2024 Apr 30;14:9950. doi: 10.1038/s41598-024-60533-5 (PMC11061191; doi:10.1038/s41598-024-60533-5)
Supplement: Supplementary file 1 — Supplementary Figure S1. [file 41598_2024_60533_MOESM1_ESM.pdf]

Supplementary Information

Circadian rhythm and surface activity in soil-dwelling caecilians (Amphibia: Gymnophiona)

Avanthika Prakash[1], David J. Gower[1,2], Ranjith Vengot[1], and Ramachandran Kotharambath[1,2,*]

[1]Dept. of Zoology, Central University of Kerala, Tejaswini Hills, Kasaragod, Kerala, India

[2] Natural History Museum, London, SW7 5BD, U.K.

[*]Corresponding author email: ram@cukerala.ac.in

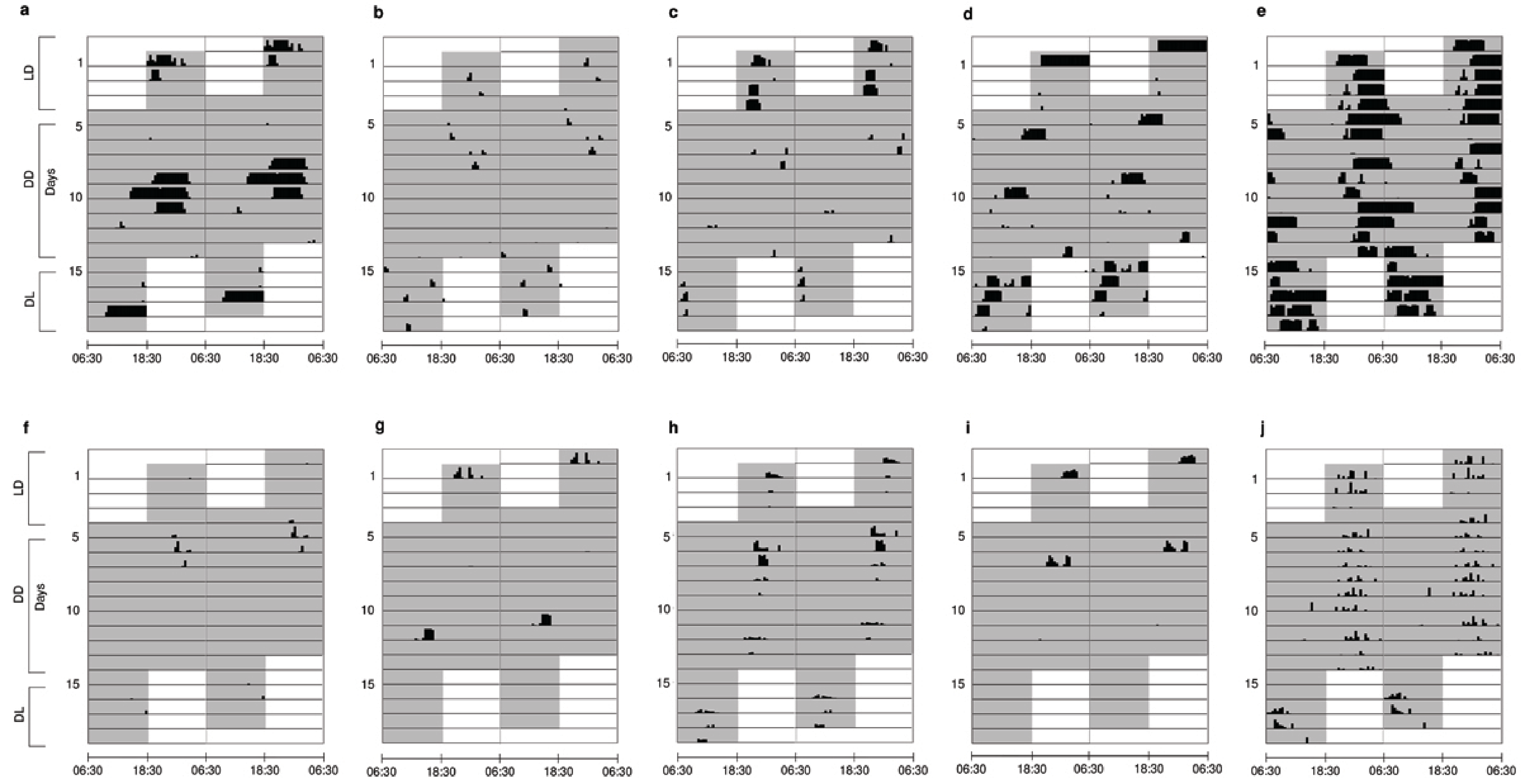

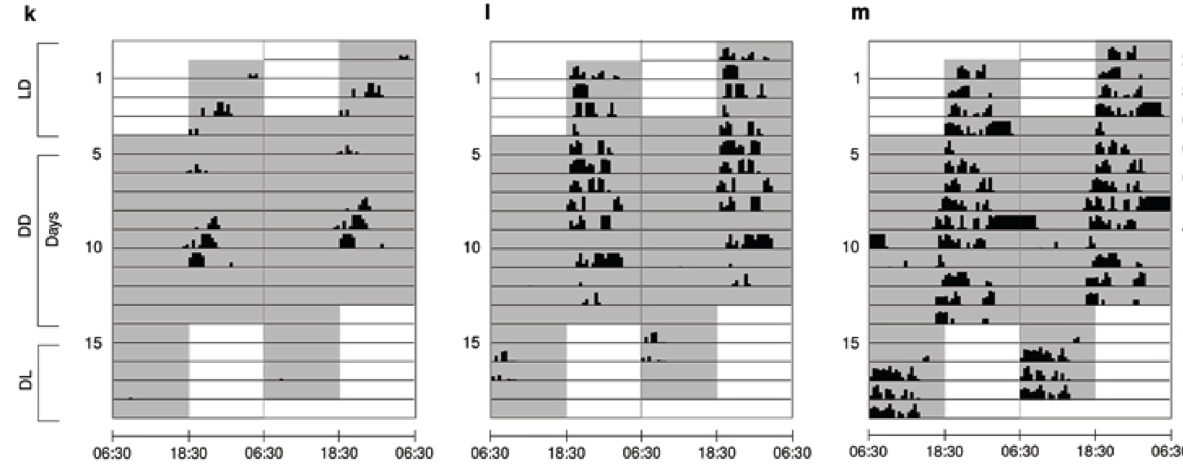

Supplementary Figure S1. Double plotted actograms represent the surface activity of 13 individuals under LD, DD and DL cycles. **(e, j, l, m)** represent surface active individuals with an evident surface activity pattern. **(a,b,c,d,f,g,h,i,k)** represent individuals with not so evident surface activity pattern. **(a-e)** represent *Uraeotyphlus* cf. *oxyurus*. **(f-j)** represent *Gegeneophis tejaswini* and **(k-m)** represent *Ichthyophis* cf. *longicephalus*. The white and gray background indicates the light and dark phases of the three cycles. Time is denoted in 24 hours.
